# Antibiotic usage in surgical prophylaxis: A prospective observational study in the surgical ward of Nekemte referral hospital

**DOI:** 10.1371/journal.pone.0203523

**Published:** 2018-09-13

**Authors:** Getachew Alemkere

**Affiliations:** Department of Pharmacology and Clinical Pharmacy, School of Pharmacy, College of Health Science, Addis Ababa University, Addis Ababa, Ethiopia; University of Utah Hospital, UNITED STATES

## Abstract

**Background:**

Surgical antimicrobial prophylaxis guidelines are considered as important interventional tools for antimicrobial resistance. Guideline compliance was poor across different countries and thus results in an inappropriate and overuse of antibiotics.

**Objective:**

To evaluate the selection, timing and duration of prophylactic antibiotic administration among surgical patients in Nekmte referral hospital.

**Method:**

Prospective, facility based cross-sectional study was conducted from 1^st^ April to 30^th^ June 2017. Data were collected using data abstraction format among surgical inpatients prescribed with surgical antibiotic prophylaxis. Surgical antimicrobial prophylaxis guidelines were used as data assessment protocols. SPSS version 21.0 was used for data entry and analysis. Descriptive statistics and binary logistic regression were used for analysis.

**Results:**

The median age of the study participants was 35.0 (IQR: 25–50) years with the preponderance (58.8%) of male patients. The median hospitalization period was 8.0 (IQR: 5–11) days. Majority of the participants were from the general surgical ward (60.1%). About 43% of the procedures were clean. Most of the surgical cases were gastrointestinal (39.2%). Only 10.6% of the drug selections comply with American Society of Health-System Pharmacists guideline. Surprisingly, none of the selections were compliant to the national Standard Treatment Guideline of the country. About 84% of the study participants received ceftriaxone. Majority of the prophylactic antibiotics (75.8%) were administered for greater than 24 hours and above half (52.3%) of the antibiotics were administered preoperatively. Emergent surgery procedures (AOR = 2.89, 95% CI: (1.09–9.10) and being a male patient (AOR = 3.10, 95% CI: 1.07–8.98) were associated with inappropriate preoperative antibiotic administration. Patients admitted to the gynecology and obstetrics ward was less likely to receive surgical prophylaxis for greater than 24 hours (AOR = 0.07, 95% CI: 0.01–0.81).

**Conclusion:**

Surgical antibiotic compliance was far below the guideline recommendation. Patients admitted in the gynecology and obstetrics ward were more likely to comply with the surgical antimicrobial prophylaxis duration recommendation. The timing was most likely to be inappropriate among male patients and patients on emergent surgery. Availability and awareness creation on the antibiotic drugs and the guidelines were important interventions recommended for appropriate surgical antimicrobial use.

## Introduction

Surgical antibiotic prophylaxis (SAP) is a very brief course of antibiotics initiated closely before the start of operative procedures to reduce postoperative surgical site infections (SSIs) [1]. SSI is one of the major complications of operative procedures and is also among the most common nosocomial infections [2]. SSI denotes a significant burden in terms of morbidity, mortality and healthcare costs [3].

Guidelines based on high-quality studies had indicated that appropriate surgical antimicrobial prophylaxis is among the effective measures for preventing SSI [4]. For optimal benefit, determining the appropriate indication, selecting agent that covers the likely pathogen on wound contamination, and administering sufficient bactericidal concentrations during the whole period that the incision is open for risk of bacterial contamination is required [4,5].

Previous studies of antibiotic prophylaxis usage have shown wide variation in compliance to guidelines [6]. Selection, timing and duration of antimicrobial prophylaxis use showed high noncompliance whereas indication and dose were relatively more satisfactory parameters [6,7]. The variation in practice across different setups could be attributed to the variation in the published guidelines, the lack of acceptance of the guideline among the surgeons, and the lack of awareness and availability of the guideline to service delivering professionals or setups [6,8].

Including our country, findings from different countries had reported that guideline compliance was poor and thus SAP was used inappropriately [7–10]. Thus, inappropriate use of surgical antibiotics has to be well-thought-out in order to achieve an overall reduction in morbidity, mortality and healthcare cost. One study done at Jimma University teaching hospital (southwestern Ethiopia) tried to assess the likely factors for poor preoperative timing and duration [10]. Another study in Ayder referral hospital (northern Ethiopia) investigated the guideline compliance regardless of assessing the contributing factors. The current study is a hybrid of the two methods in that it not only addresses the guideline compliance but also tried to assess the likely factors contributing to guideline non-adherence for drug selection, preoperative timing and duration of SAP.

The assessment of SAP utilization was done in comparison to the guidelines in the area. Since there was no local data like antibiogram, institutional resistance patterns or guideline prepared by the hospital, adherence was assessed based on the country’s national Standard Treatment Guideline (STG) [5] and the American Society of Health-System Pharmacists (ASHP) guideline [4].

This study was done to identify gaps and set appropriate recommendations to improve future utilization of surgical antimicrobial prophylaxis. Hence it was aimed at evaluating the selection, timing and duration of prophylactic antibiotic administration among surgical patients in Nekmte Referral Hospital (NRH). The study can be used for guiding the hospital decision on surgical antibiotic usage and for policy direction. It can also be inferred for future surveys in similar setups.

## Methods

### Study setting and period

The study was conducted in the surgical wards of NRH during the period from 1^st^ April to 30^th^ June 2017, for three consecutive months. Nekmte is located at 331 km to west of Addis Ababa. The hospital has different departments and wards like the outpatient department (OPD), medical wards, gynecology and obstetrics ward, pediatrics ward and general surgical wards. The general surgical ward has 49 beds with a 14 bed separated orthopedic ward. Almost all the recording systems of the hospital during the study period were carried out manually.

### Study design

A 3-month long facility based cross-sectional study was employed to assess SAP usage.

### Study population

All patients attending the gynecologic and obstetrics, general surgical and orthopedic wards during the study period were considered as the study population and those that fulfill the inclusion criteria were included.

### Inclusion criteria

Using a convenient sampling method 153 adult patients who underwent surgical procedures in NRH during the study period were addressed based on the following criteria.

Inclusion criteria

- Adult surgical patients
- Clean, clean-contaminated and contaminated procedures
- Prophylactic antibiotic use
- General surgical, orthopedic and gynecology wards

Exclusion criteria

- Pediatrics (<18 years old)
- Dirty procedures
- Therapeutic and other non-surgical prophylaxis uses
- Emergency OPD and emergency obstetrics wards
- Presence of infection and/or antibiotics before surgery

### Study variables

#### Dependent variables

- Compliance with surgical antibiotic usage to the national and International guidelines

#### Independent variables

- Socio-demographic factors like sex and age
- Patient medical conditions
- Perioperative characteristics
- Antibiotic regimens
- Demographics of antibiotic providers

### Data collection process

Data abstraction format was prepared based on previously published studies [7,9,10]. It was pretested on 15 patients, modified accordingly and used for data collection. The data abstraction format was divided into five sections. (1) Patient demographic and medical data, (2) surgical data (class of surgery, type of surgery, time of incision, shift surgery is done, duration of surgery, wound class, type of ward and length of hospital stay), (3) SAP usage data (antibiotic name, dose, frequency, preoperative administration time relative to incision and duration of prophylactic administration), (4) surgical antibiotic providers data (provider profession, sex, age and experience). Then finally (5) appropriate SAP usage assessment lists (indication, selection, duration, and timing) were noted. Based on this, all relevant data were collected from patients' medical records. After data collection, however, there are some important variables that were excluded from the analysis because of incomplete recording on the patient cards and/or inconsistent data abstraction from the patient medical record. These were relevant comorbidities, re-dose timing, and post-surgical infection status. Wound classification was performed with the consultation of a pre-informed surgeon. Data were collected by four trained nurses working in the hospital, under the supervision of the principal investigator.

### Data interpretation

The CDC wound classification [11] was used to categorize operative procedures into clean, clean-contaminated and contaminated. The appropriateness of SAP use was assessed against national STG and one of the most internationally recognized reputable guideline, ASHP [4,5]. The two guidelines, in fact, differ in some major respects. For instance, the ASHP guideline independently presents evidenced-based recommendations on antimicrobial usage in surgery; however, the Ethiopian STG presented a brief summary of ‘antimicrobial usage in surgery' as a subtopic under its first chapter and it is also poor in evidencing its recommendation. They also have overlapping recommendations. Supplementary data on the recommendation of the two guidelines for the major surgical categories is available as supporting information [S1 File]. The appropriateness was evaluated with regard to the necessity for SAP use (indication), choice of antibiotic (selection), preoperative timing (timing) and total duration of prophylaxis (duration). If more than one drug was given, the regimen as a whole is considered for evaluation. Thus any deviation from the guideline of one or both of the antibiotics would lead to a final report of noncompliance with the guidelines recommendation. If an antibiotic was given while it was not indicated, the parameter of antibiotic selection was not evaluated. Regardless of this the timing and duration compliance were assessed for all antibiotics administered. The timing of administration was analyzed as in the intervals before and after incision.

### Statistical analysis

The collected data for any deficit was checked and cleaned prior to data entry. Data were coded, cleaned, entered and analyzed with SPSS for Windows version 21.0. Descriptive statistics, such as medians, interquartile ranges (IQR) and percentages were used to summarize patient characteristics. Factors affecting compliance were assessed using a binary logistic regression model. Those variables that had an association (p<0.05) in the univariate model were entered into the final multiple bin ary logistic regression models. The odds ratio was used to report the statistical association. The association was declared at P<0.05.

### Operational definitions

The surgical antibiotic administration was considered (1) early preoperative administration if given earlier than 60 minutes before skin incision, (2) preoperative if within 60 minutes before skin incision, (3) perioperative if within 3 hours after skin incision and (4) postoperative if after 3 hours after skin incision. SAP selection was said to be (1) adequate/compliant: if the drug/s were namely recommended by the guideline, (2) narrow: when only one of the drug recommended by the guideline is used, and (3) broader: when extra drug is added to the guideline recommendation (4) unrelated: if the drug name is not part of the recommendation list regardless of its relatedness to the recommended list or regardless of its spectrum of coverage.

### Ethical consideration

Ethical approval for the protocol was obtained from the Ethics Committee of the Department of Pharmacy, College of Health Sciences, Wollega University. In addition, the hospital management was requested officially for permission to conduct the study. Furthermore, the research was undertaken after receiving oral consent from the patients and the health professionals attending the patient during the study period. To ensure confidentiality, name and other identifiers of patients and prescribers were not recorded on the data abstraction instruments. The collected data was kept in a locked cabinet and only the researchers had access.

## Result

### Socio-demographic characteristics of study participants

Medical records of 153 surgical patients administered with a prophylactic antibiotic were followed and evaluated. The median age of the study participants was 35.0 (IQR: 25–50) years. Males account for 58.8% of the study participants. The median hospitalization period was 8.0 days (IQR: 5–11 days). About 97% of the patients were discharged with improvement. Majority of the participants were from the general surgical ward (60.1%) **(Table 1)**.

**Table 1:** Characteristics of surgical inpatients taking SAP in NRH from 1^st^ April to 30^th^ June 2017.

Variable	Frequency (N = 153) (%)
**Age** (Median (Interquartile range)	35.0 (25–50)
**Sex**	
Male	90 (58.8)
Female	63 (41.2)
**LoS (**Median (Interquartile range)**)**	8.0 (5–11)
**Final status**	
Improved	149 (97.4)
Dead	4 (2.6)
**Ward**	
General surgical	92 (60.1)
Gynecology-obstetrics	38 (24.8)
Orthopedic	23 (15.0)

### SAP provider demographics

Antibiotic prophylaxis was administered by nurses in all except one. The majority of them (76.5%) were females. Above half of the providers were in the age range of 30–40 years and most of them had an experience of 8–10 years **(Table 2)**.

**Table 2:** Socio-demographic characteristics of the surgical prophylaxis providers in NRH from 1^st^ April to 30^th^ June 2017.

Age of provider	Frequency (n = 153) (%)
< 30 years	45 (29.4)
30–40 years	81 (52.9)
> 40 years	27 (17.6)
**Sex of provider**	
Male	36 (23.5)
Female	117 (76.5)
**Experience of provider**	
8–10 years	91 (59.5)
> 10 Years	62 (40.5)

### Perioperative characteristics of surgical patients

Most of the surgical cases were gastrointestinal (39.2%) followed by gynecology and obstetrics (15.7%). Majority of the procedures were clean (40.7%), followed by clean-contaminated (32%). Thirteen (8.5%) patients had a urinary catheter. Most of the surgical procedures took 1–2 hours (56.2%) and about 67% of the procedures were performed in the morning. Sixty-eight (44.4%) of the patients took antibiotics for 2–5 days and less than a quarter (20.9%) of the patients took for not greater than a day. About 80% of the antibiotics were started before surgical incision and above half of the patients took preoperatively, within one hour of surgical incision **(Table 3)**.

**Table 3:** Preoperative characteristics of study participants at NRH from 1^st^ April to 30^th^ June 2017.

Perioperative characteristics	Frequency (%)
**Class of Surgery**	
Gastrointestinal	60 (39.2)
Gynecology and obstetrics	38 (24.8)
Orthopedic	24 (15.7)
Urologic	16 (10.5)
Head and neck	3 (2.0)
Others	12 (7.8)
**Wound Class (n = 153)**	
Clean	66 (43.1)
Clean-contaminated	49 (32.0)
Contaminated	38 (24.8)
**Presence of Catheter**	
Yes (Catheter (13)	13 (8.5)
No	140 (91.5)
**Duration of surgery (hours) (n =** 153)	
< 1	66 (43.1)
1–2	86 (56.2)
> 2	1 (0.7)
**Shift surgery done**	
Morning	103 (67.3)
After	20 (13.1)
Before mid-night	26 (17.0),
After mid-night	4 (2.6)
**Duration of prophylaxis administration (days)**	
One day	32 (20.9)
2–5 days	68 (44.4)
6–7 days	34 (22.2)
8–14 days	15 (9.8)
>15 days	4 (2.6)
**Timing of prophylaxis**	
Before incision	122 (79.7)
After incision	31 (20.3)
**Timing compliance with the intervals (n = 153)**	
Early	42 (27.5)
Preoperation	80 (52.3)
Perioperative	20 (13.1)
Postoperative	11 (7.2)

### Surgical type and procedures

Sixty (39.2%) surgical cases were gastrointestinal. Appendicitis is the most common gastrointestinal diagnosis (19/60), followed by colorectal cases (17/60) making an appendectomy the most frequently performed gastrointestinal procedure. Among the 38 gynecology and obstetrics case, 33 were gynecologic only (17 utero-vaginal prolapses). Out of 24 orthopedic procedures, 23 were different types of fractures. Eight external fixations and seven open reduction and internal fixations (ORIF) were the most commonly performed orthopedic procedures. Among the 16 urologic procedures, 12 were Prostatectomy followed by 3 hydrocelectomy procedures **(Table 4)**.

**Table 4:** Surgical type and procedures of surgical inpatients at NRH from 1^st^ April to 30^th^ June 2017.

Surgery type (F)	Diagnosis (F)	Procedure (F)	F (%)
**GI**			**60 (39.2)**
Gastro-duodenal/ General (11)	Perforated abdomen (4),	Repair with Graham’s Patch (4)	
	Penetrating abdomen (2), blunt abdominal trauma (2)	Laparotomy (4)	
	Mesenteric cyst (1)	Excision (1)	
	Gastric outlet obstruction (1), Post-operative adhesion (1)	Gastrojejunostomy (1), Repair (1)	
Biliary Tract (3)	Cholelithiasis (2), gallbladder stone (1)	Cholecystectomy (2), Laparotomy (1)	
Appendectomy (19)	Appendicitis (19)	Appendectomy (19)	
Small bowel (7)	Small bowel obstruction (6),	R+A (6),	
Hernia (4)	Hernia (4)	Herniorrhaphy (4)	
Colorectal (17)	Large bowel obstruction (8),	R+A (6), laparotomy (2),	
	Colostomy (4),	Colostomy closure (2), Permanent colostomy (2)	
	Rectal Cancer (2), hemorrhoid (2), Perianal fistula (1)	Permanent colostomy (1), Hemorrhoidectomy (2), Fistulectomy (1)	
**Gynecology and obstetric**	Utero-vaginal prolapse (17)	Vaginal Hysterectomy (17)	**38 (24.8)**
	Myoma (9), Endometrial cancer (1)	Myomectomy (8), Total Abdominal Hysterectomy (2)	
	Adnexal Cyst/Tumor (6),	Salpingectomy (4), laparotomy (1), Cystectomy (1)	
	Antepartum hemorrhage /Uterine Rupture(5)	Laparotomy (2), TAH (2), Bilateral tubal ligation (1)	
**Orthopedic surgery**	Fracture (23)	Debridement (3), External Fixation (8), Gator (3), ORIF (6), TBW (3)	**24 (15.7)**
	Gangrene (1)	Amputation	
**Urologic surgery**			**16 (10.5)**
	BPH (12)	Prostatectomy (12)	
	Hydrocele (3)	Hydrocelecectomy (3)	
	Hydronephrosis (1)	R+A (1)	
**Head and neck**			**3 (2.0)**
	Goiter (3)	Thyroidectomy (3)	
**Others surgeries**			**12 (7.8)**
➢ Skin and deep tissue (5)	Skin cancer (1)/ Lipoma (1)	Excision (2)	
	Fasciitis (1)/ Malunion (1)/Soft tissue injury (1)	Fasciectomy (1), skin graft (1), repair (1)	
➢ Breast (2)	Breast mass/cancer	Mastectomy (2),	
➢ Miscellaneous	Pelvic mass (1), Popliteal cysts (1),	Excision (2)	
	wound dehiscence (1)	Wound Closure	
	Stab injury (1), animal bite (1)	Laparotomy (2)	
**Total**			**153 (100)**

F: frequency, TAH: Total Abdominal Hysterectomy, R & A: resection and anastomosis, ORIF: open reduction and internal fixations, TBW: tension banding and wiring.

### Antibiotics used for prophylaxis

About 59% of patients took a single prophylactic drug and about 39% took a combination of two drugs. Namely, about 84% of the participants received ceftriaxone. Metronidazole (35.3%) is the second most prescribed prophylactic antimicrobial followed by ampicillin (19.6%) **(Table 5)**.

**Table 5:** Utilization pattern of SAP among surgical patients at NRH from 1^st^ April to 30^th^ June 2017.

Variables	Frequency (n = 153) (%)
**Number of prophylactic antibiotic(s) used (n = 153)**	
One	90 (58.8)
Two	60 (39.2)
Three	3 (2.0
**Name of Prophylactic antibiotics used**	
Amoxicillin	1 (0.7)
Ampicillin	21 (13.7)
Cloxacillin	2 (1.3)
Ceftriaxone	66 (43.1)
Ceftriaxone + Ampicillin	6 (3.9)
Ceftriaxone + Ampicillin + Metronidazole	3 (2.0)
Ceftriaxone + Metronidazole	51 (33.3)
Ceftriaxone + Cloxacillin	3 (2.0)
**Specific prophylactic antibiotic used per patient**	
Ceftriaxone	129 (84.3)
Ampicillin	54 (35.3)
Metronidazole	30 (19.6)
Cloxacillin	5 (3.3)
Amoxicillin	1 (0.7)
**Prophylactic antibiotic use duration (n = 153)**	
Median (interquartile range) (days)	5.0 (3–7)

### SAP compliance to the guidelines

About 20% of the prophylactic drugs were given for cases that lack specific recommendation as per the ASHP guideline. Among the remaining (80.4%) recommended indications, only 10.6% of the selections were adequate/compliant with the guideline recommendation. Majority of the (67.5%) selections were unrelated to the recommendations and 19.5% were unnecessarily broader than the guideline recommendations. On the other hand, all of the administrations were non-concordant to the Ethiopian national STG for general hospitals **(Table 6)**.

**Table 6:** SAP compliance to national STG and ASHP guidelines among surgical patients in NRH from 1^st^ April to 30^th^ June 2017.

SAP compliance (n = 153)	STG		ASHP	
**Indication compliance**	**Frequency**	**%**	**Frequency**	**%**
Given with Indication	126	82.4	123	80.4
Given without indication	27	17.6	30	19.6
**Selection compliance**	**(n = 126)**		**(n = 123)**	
Adequate/concordant	0	0	13	10.6
Narrow	0	0	3	2.4
Broader	42	33.3	24	19.5
Unrelated	84	66.7	83	67.5

**Fig 1:**
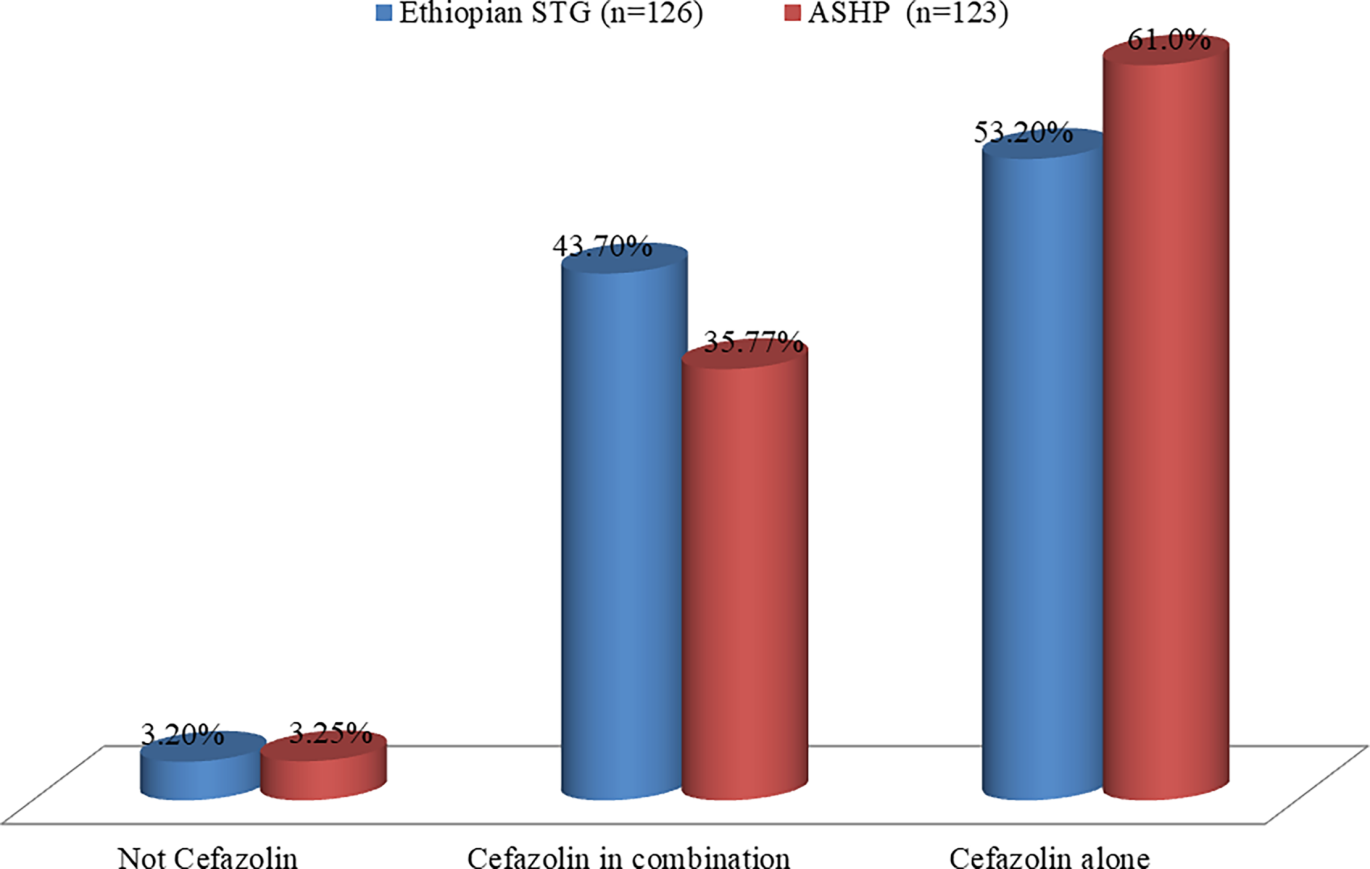
Number of cases that can be addressed as per the guidelines if cefazolin is available among surgical patients in NRH from 1^st^ April to 30^th^ June 2017. STG: standard treatment guideline; ASHP: American society of health-system pharmacists.

If cefazolin is available more than half of the cases (67 (53.2%) of STG and 75 (61.0%) of ASHP) will be managed with cefazolin alone as per the guideline recommendations. In combination with other antibiotics, cefazolin can further be the option for 55 (43.7%) of the cases as per the STG and 44 (35.8%) of the cases as per the ASHP guideline recommendations. Generally, about 97% of the cases need cefazolin as per the guideline recommendations (**Fig 1**).

### SAP duration and preoperative timing

Majority of the prophylactic antibiotics were administered for greater than 24 hours (75.8%) and about 48% of the administrations were not given in the appropriate preoperative period of within 0 to 60 minutes before incision as recommended by the guidelines (52.3) **(Table 7)**.

**Table 7:** Duration and timing of SAP among surgical inpatients in NRH from 1^st^ April to 30^th^ June 2017.

Variable	Frequency (n = 153)	%
Duration less or equal to 24 hours		
Yes	37	24.2
No	116	75.8
Timing within 0 to 60 minutes prior to incision		
Yes	80	52.3
No	73	47.7

### Factors affecting timing and duration of SAP

#### Factors affecting the timing of SAP administration

In the univariate model sex of the patient, surgery type, ward, wound class, duration of prophylaxis administration, and sex and experience of the provider does have an association with preoperative SAP administration timing at a p-value of less than 0.05 **(Table 8)**.

**Table 8:** The univariate analysis of the factors affecting the timing of SAP among surgical inpatients in NRH from 1^st^ April to 30^th^ June 2017.

Variables	Timing (not within 60 minutes before incision)	COR (95% C.I.)	Sig.
Sex of patient (Male)	55 (61.1)	3.93 (1.97, 7.85)	0.000
Age of patient		1.01 (0.99, 1.03)	0.563
Ward			
Surgical	52 (56.5)	1.00 (0.40, 2.51)	1.000
Genecology and obstetric	8 (21.1)	0.21 (0.07, 0.64)	0.006
Orthopedic	13(56.5)	[Reference]	
Surgery type (Emergent)	44 (57.9)	2.28 (1.19, 4.35)	0.013
Wound class			
Clean	35(53.0)	0.74 (0.33, 1.66)	0.459
Clean-contaminated	15 (30.6)	0.29 (0.12, 0.70)	0.006
Contaminated	23 (60.5)	[Reference]	
Duration of surgery		0.99 (0.99, 1.01)	0.703
Presence of medical device		1.64 (0.52, 5.14)	0.399
24 hour and less SAP duration	10 (27)	0.31 (0.14, 0.70)	0.005
Sex of the provider (Male)	27 (75.0)	4.63 (1.99, 10.73)	0.000
Age of the provider			
Age (<3o years)	12 (26.7)	0.83 (0.026, 0.27)	0.000
Age (30–40 years)	39 (48.1)	0.21 (0.07, 0.61)	0.004
> 40 years	22 (81.5)	[Reference]	
Experience of provider (≤ 10 years)	35 (38.5)	0.40 (0.20, 0.77)	0.006

Using those variables that had a p value of less than 0.05 in the univariate analysis emergent surgery procedures (AOR = 2.89, 95% CI: (1.09–9.10) and being a male patient (AOR = 3.10, 95% CI: 1.07–8.98) were the two factors that were independently associated with the inappropriate timing of a prophylactic administration as per the guidelines **(Table 9)**.

**Table 9:** The multivariate analysis of the factors that determine the timing of SAP administration among surgical patients in NRH from 1^st^ April to 30^th^ June 2017.

Variables	Timing (not within 60 minutes before incision)	AOR (95% C.I.)	Sig.
Sex (Male)	55 (61.1)	3.10 (1.07, 8.98)	0.037
Ward			
Surgical	52 (56.5)	1.11(0.32, 3.83)	0.873
Genecology and obstetric	8 (21.1)	0.76 (0.06, 8.79))	0.823
Orthopedic	13(56.5)	[Reference]	
Surgery type (Emergent)	44 (57.9)	2.89 (1.09, 9.10)	0.049
Wound class			
Clean	35(53.0)	1.66 (0.54, 5.08)	0.376
Clean-contaminated	15 (30.6)	0.69 (0.21, 2.23)	0.525
Contaminated	23 (60.5)	[Reference]	
24 hour and less SAP duration	10 (27)	0.55 (0.18, 1.69)	0.292
Sex of the provider (Male)	27 (75.0)	1.52 (0.36, 6.30)	0.568
Age of the provider			
Age (<3o years)	12 (26.7)	0.31 (0.03, 3.03)	0.312
Age (30–40 years)	39 (48.1)	0.17 (0.02, 1.220	0.078
> 40 years	22 (81.5)	[Reference]	
Experience of provider (≤ 10 years)	35 (38.5)	1.40 (0.53, 3.67)	0.494

#### Factors associated with greater than 24 hour SAP use duration

In the univariate model sex of the patient, surgery type, ward, length of stay and sex and experience of the provider does have an association with prolonged prophylaxis administration at a p-value of less than 0.05 (**Table 10)**.

**Table 10:** The univariate analysis of the factors attributing to a long duration of SAP use among surgical inpatients in NRH from 1^st^ April to 30^th^ June 2017.

Variables	> 24 hour SAP duration, frequency (%)	COR (95% C.I.)	Sig.
Age		1.00 (0.98, 1.02)	0.991
Sex (Male)	82 (91.1)	8.74 (3.63, 21.06)	0.001
Surgery type (emergent)	66 (86.8)	3.56 (1.58, 8.04)	0.002
Ward			
Surgical	86 (93.5)	3.02 (0.78, 11.75)	0.11
Genecology & obstetric	11 (28.9)	0.09 (0.02, 0.31)	0.000
Orthopedic	19 (82.6)	[Reference]	
Duration of surgery		0.99 (0.98, 1.01)	0.746
Length of stay (< 8 days)	63 (66.3)	0.19 (0.07, 0.51)	0.001
Sex of Provider(Male)	32 (88.9)	3.14 (1.03, 9.58)	0.044
Experience (≤ 10 years)	61 (67.0)	0.26 (0.11, 0.64)	0.003
Age of provider			
< 30 years	18 (45)	0.12 (0.13, 1.39)	0.231
30–40 years	75 (81)	2.17 (0.56, 8.37)	0.259
> 40 years	23 (27)	[Reference]	

Using those variables that had a p value of less than 0.05 in the univariate analysis, only ward of admission had an independent association with SAP use duration. Patients admitted to gynecology and obstetrics ward were more likely to receive SAP for less than or equal to 24 hour days (AOR = 0.07, 95% CI: 0.01–0.81) than patients admitted to orthopedic ward **(Table 11)**.

**Table 11:** The multivariate analysis of the factors associated with prolonged SAP use duration among surgical patients in NRH from 1^st^ April to 30^th^ June 2017.

Variables	> 24 hour SAP use duration	AOR (95% C.I.)	Sig.
Sex (Male)	82 (91.1)	1.01 (0.18, 5.52)	0.994
Surgery type (emergent)	66 (86.8)	1.14 (0.33, 3.94)	0.839
Ward			
Surgical	86 (93.5)	2.82 (0.62, 12.81)	0.178
Genecology & obstetric	11 (28.9)	0.07 (0.01, 0.81)	0.033
Orthopedic	19 (82.6)	[Reference]	
Sex of Provider(Male)	32 (88.9)	1.18 (0.29, 4.88)	0.817
Length of stay (< 8 days)	63 (66.3)	0.78 (0.22, 2.80)	0.702
Experience (≤ 10 years)	61 (67.0)	1.94 (0.44, 8.50)	0.378

## Discussion

In this study, the median hospitalization period of the study participants was 8.0 days (IQR 5–11 days) and about 97% of the patients being discharged with improvement (Table 1). Most of the surgical cases were gastrointestinal (39.2%) followed by gynecology and obstetrics (15.7%). Majority of the procedures were clean (40.7%) followed by clean-contaminated (32%). Thirteen (8.5%) patients had a urinary catheter. Most of the surgical procedures took 1–2 hours (56.2%) and about 67% of the procedures were performed in the morning (Table 4).

In our study, nurses administered the antibiotic prophylaxis in all but one case, in contrast to a similar Palestinian study where nurses account 35.5% [9]. The majority of nurses were females (76.5%) and all had 8 or more years of experience. Although not seen in this study, the Palestine study had shown the association of the health providers' variables with appropriate surgical prophylaxis usage [9].

Similar to studies in Ayder referral hospital (19.4%) [7] and in Nicaragua (23%) [12], in this study, 19.6% of the prophylactic antibiotics were given without indication as per the ASHP guideline [4,5]. Among the remaining (80.4%) recommended indications, only 10.6% of the selections were adequate/compliant to the ASHP guideline recommendation. Majority of the (67.5%) administrations were unrelated to the recommendations and 19.5% were unnecessarily broader than the guideline recommendations. On the other hand, all of the prophylactic selections were non-concordant to the Ethiopian national STG for general hospitals. Similar evidence is noted from Ayder referral hospital report [7].

Ceftriaxone was excessively and inappropriately used for SAP in our study site, NRH. Comparable to Ayder study [7] about 84% of the participants were given ceftriaxone despite the drug is not mentioned in the national STG of the country. As per the ASHP, ceftriaxone is recommended only for high-risk biliary tract procedures and for colorectal surgery. This is because being a broad spectrum drug ceftriaxone can induce emergency of resistance more likely than cefazolin and other widely used surgical prophylactic drugs.

Of the patients (n = 123) to whom surgical prophylaxis was indicated and administered; 89.4% (67.5% unrelated to the recommendations, 19.5% broader and 2.4% narrower than the guideline recommendations) of the selections were inappropriate. This supports the study of Ayder (north Ethiopia) (89.5%) [7] and Iran (92.5%) [13] but higher than studies from Sudan (64.4%) [14], Qatar (31.5%) [15] and Nicaragua (66.8%) [12].

In this study, one of the reported reasons for noncompliance was the use of agents that were unrelated to the SAP guideline recommendation. In addition, about 20% of the cases contain extra combinational drugs on the recommended regimen. Most of the unrelated cases were also much broader than the recommended prophylactic agent or had an unnecessary combination. Probably this may be because of the wrong generalization that broad/combination antibiotics are more effective in averting SSIs.

This research finding indicated that surgical antibiotics commonly practiced in NRH were neither part of the STG of the country nor the primary choice of the international guidelines. Analogous reports were noted in similar studies conducted in different areas of the country [7,10]. Therefore, what one should be skeptical to our country context is that some common custom on broad-spectrum antibiotic use has been held and communicated across (probably) all hospitals of the country that should be further investigated and intervened as soon as possible. Probably, another factor to be investigated in line with this issue is the availability of the recommended drugs and the reasons behind in the given facility. For instance, in this case, despite cefazolin is available in the country, the NRH drug list does not contain it. If cefazolin was available it can be one of the options for about 97% of the surgical cases as per the guideline recommendation. In support of this, a study from Jordan [16] found that 68.1% of inappropriate antimicrobial selections for surgical prophylaxis were attributed to drug unavailability.

Despite the availability of first choice drugs, surgeons had been reported to fail to comply with the guideline recommendations. Some of the reasons mentioned as a barrier to adherence to the guideline were lack of agreement of surgeon’s with guideline recommendations, lack of awareness of appropriate guidelines, and logistic limitations in the surgical wards [8].

Another common failure to guidelines compliance investigated in this study was prolonged surgical prophylaxis administration duration beyond the recommendation. Although the guidelines promote to end prophylactic administration within 24 hours [4,5], 44.4% of the administrations continued for up to five days. Comparable results were reported from a study in Iran (46%) [13], and in southwestern Ethiopia (50.7%) [10]. To be specific, in this study only less than a quarter (20.9%) of the patients’ discontinued surgical antibiotics within a day. About 79% of the remaining antibiotics were given for greater than a day, without any reason. This result is comparable with studies in Nicaragua (78.4%) [12] and Sudan (97%) [14], but much higher than a study in Malaysia (20%) [17]. Guidelines basing on high-quality studies identified that prolonged use of antimicrobials may contribute to bacterial resistance; urging our setup to reconsider guideline-concordant use [4].

In this study, another area of focus that needs improvement as per the guideline recommendation was initial (preoperative) timing of surgical antimicrobial prophylaxis. About 48% of the prophylactic administrations were not given in the preoperative time range (within 60 minutes of surgical incision). Prophylactic antibiotics are more effective if administered as close as to skin incision time; especially within one hour before skin incision [4,5]. In this study, only 80 (52.3%) patients got administered within 60 minutes before skin incision. A similar finding was obtained from studies of Jimma University Specialized Hospital (56%) [10] and India [18]

Among the three factors assessed for guideline compliance, an inappropriate selection was not considered for association test because all of the selections are inappropriate as per the national STG and only colorectal surgery selections are found appropriate as per the ASHP guideline. The remaining two variables, duration, and preoperative timing were assessed. As a result, patients admitted to gynecology and obstetrics ward were more likely to receive prophylaxis for less than or equal to 24 hours (AOR = 0.07, 95% CI: 0.01–0.81) than patients admitted to the orthopedic ward. This finding was shared with a study done in the west bank, Palestine [9], in which case gynecology and obstetrics department had much better adherence to the antibiotic duration guideline. On the other hand, emergent surgery procedures (AOR = 2.89, 95% CI: 1.09–9.10) and being a male patient (AOR = 3.10, 95% CI: 1.07–8.98) were the two factors that were associated with the inappropriate preoperative timing of a prophylactic administration. Emergency procedures may put the health workers under psychological or empathic stress. In addition, multiple care requirements in emergent procedures may probably result in poor timing. This implies that these patients should be the focus of care and the health professionals working in this area should be adequately trained on how to handle emergency conditions. We were unable to identify other studies where sex is associated with rate of antibiotic compliance, and potential reasons for this are worth future investigation.

Despite this study is a three-month-long institution based cross-sectional study, the findings should be interpreted in light of the resultant limitations. Primarily, since it is a conveniently sampled observational study, selection bias may be introduced during sampling. In addition misclassification of the wound type may also be likely. Furthermore, the study was performed in an institution with a relatively small sample size. This would limit the generalizability of the study to other areas. In addition, the current study did not consider some important parameters of SAP namely route, dose and redosing. The dose and the route of administration might not be as such critical because these issues were usually more concordant with the guideline recommendations in our country context too [7]. Redosing might be not critical in this study where ceftriaxone was the most used drug with a long half-life. Although the use of multivariate analysis helped to control substantial confounding variables, data related to the baseline characteristics, such as medical comorbidities (including diabetes, obesity etc), body mass index, medications (like steroids, immunosuppressants), baseline nutritional status, smoking status etc. were not reported in his study. These are known risk factors for infection and could influence provider decisions regarding antibiotic prophylaxis regimen. Furthermore, most of the data were retrieved from the medical records in which the accuracy of the data depends on the recording quality. Therefore, all these might have affected the outcomes and thus future studies should take these variables into consideration.

## Conclusion

In conclusion, the selection and duration of prophylactic antibiotic administration were far below the guideline recommendation but the preoperative timing was about half times compliant [S6 and S7 Tables]. The timing of surgical antimicrobial prophylaxis was most likely to be inappropriate for male patients and patients in emergent surgery [S9 Table]. Patients admitted to the gynecology and obstetrics ward were more likely to comply with the antimicrobial duration recommendation [S11 Table]. Availability and awareness creation on the antibiotic drugs and the guidelines were important interventions for appropriate surgical antimicrobial use. Therefore, healthcare administration and other concerned authorities should work to improve the availability of antibiotics and ensure compliant use in accordance with guidelines. Future researches should focus on addressing easily modifiable and worthwhile issues to the clinical practice like availability of recommended antibiotics in the given setting and physician reasons for antibiotic selection.

## Supporting information

S1 Table Characteristics of surgical inpatients taking SAP in NRH from 1^st^ April to 30^th^ June 2017. (DOCX)

S2 Table Socio-demographic characteristics of the surgical prophylaxis providers in NRH from 1^st^ April to 30^th^ June 2017. (DOCX)

S3 Table Preoperative characteristics of study participants at NRH from 1^st^ April to 30^th^ June 2017. (DOCX)

S4 Table Surgical type and procedures of surgical inpatients at NRH from 1^st^ April to 30^th^ June 2017. F: frequency, TAH: Total Abdominal Hysterectomy, R & A: resection and anastomosis, ORIF: open reduction and internal fixations, TBW: tension banding and wiring.
(DOCX)

S5 Table Utilization pattern of SAP among surgical patients at NRH from 1^st^ April to 30^th^ June 2017. (DOCX)

S6 Table SAP compliance to national STG and ASHP guidelines among surgical patients in NRH from 1^st^ April to 30^th^ June 2017. (DOCX)

S7 Table Duration and timing of SAP among surgical inpatients in NRH from 1^st^ April to 30^th^ June 2017. (DOCX)

S8 Table The univariate analysis of the factors affecting the timing of SAP among surgical inpatients in NRH from 1^st^ April to 30^th^ June 2017. (DOCX)

S9 Table The multivariate analysis of the factors that determine the timing of SAP administration among surgical patients in NRH from 1^st^ April to 30^th^ June 2017. (DOCX)

S10 Table The univariate analysis of the factors attributing for long duration of SAP use among surgical inpatients in NRH from 1^st^ April to 30^th^ June 2017. (DOCX)

S11 Table The multivariate analysis of the factors associated with prolonged SAP use duration among surgical patients in NRH from 1^st^ April to 30^th^ June 2017. (DOCX)

S1 File The ASHP and Ethiopian STG SAP recommendations for each surgical category. (DOCX)

## References

[1] Waddell TK, Rotstein OD. Antimicrobial prophylaxis in surgery. Committee on Antimicrobial Agents, Canadian Infectious Disease Society. CMAJ. 1994;151(7):925–31. 7922928 PMC1337278

[2] Wenzel RP. Health Care-Associated Infections: Major Issues in the Early Years of the 21^st^ Century. Clin Infect Dis [Internet]. 2007;45(Supplement_1): S85–8. Available from: http://academic.oup.com/cid/article/45/Supplement_1/S85/358288/17582577 10.1086/518136

[3] Perencevich EN, Sands KE, Cosgrove SE, Guadagnoli E, Meara E, Platt R. Health and economic impact of surgical site infections diagnosed after hospital discharge. Emerg Infect Dis. 2003;9:196–203. 10.3201/eid0902.020232 12603990 PMC2901944

[4] Bratzler DW, Dellinger EP, Olsen KM, Perl TM, Auwaerter PG, Bolon MK, et al Clinical Practice Guidelines for Antimicrobial Prophylaxis in Surgery. Best Pract Hosp Heal Pharm. 2013;70(3):582–667. 10.2146/ajhp120568 23327981

[5] FMHACA. Standard Treatment Guidelines For General Hospital: Good Prescribing & Dispensing Practices for Better Health Outcomes. third edit. Addis Ababa; 2014. 13–15 p.

[6] Ng RS, Chong CP. Surgeons’ adherence to guidelines for surgical antimicrobial prophylaxis—A review. Australas Med J. 2012;5(10):534–40. 10.4066/AMJ.2012.1312 23173017 PMC3494825

[7] Mohamoud SA, Yesuf TA, Sisay EA. Utilization Assessment of Surgical Antibiotic Prophylaxis at Ayder Referral Hospital, Northern Ethiopia. J Appl Pharm. 2016;08(02).

[8] van Kasteren MEE, Kullberg BJ, de Boer AS, Mintjes-de Groot J, Gyssens IC. Adherence to local hospital guidelines for surgical antimicrobial prophylaxis: A multicentre audit in Dutch hospitals. J Antimicrob Chemother. 2003;51(6):1389–96. 10.1093/jac/dkg264 12746377

[9] Musmar SM, Ba’Ba H, Owais A. Adherence to guidelines of antibiotic prophylactic use in surgery: a prospective cohort study in North West Bank, Palestine. BMC Surg. 2014;14(69):1–7. 25204205 10.1186/1471-2482-14-69 PMC4168988

[10] Jisha H. Timing of Prophylactic Antibiotic Administration in Elective Surgical Patients at Jimma University Teaching Hospital: South West Ethiopia. J Anesth Clin Res. 2016;07(04):1–7.

[11] Mangram AJ, Horan TC, Pearson ML, Silver LC, Jarvis WR. Guideline for Prevention of Surgical Site Infection, 1999. Infect Control Hosp Epidemiol. 1999;20(04):247–80. 10.1086/501620 10219875

[12] van Disseldorp J, Slingenberg EJMH, Matute A, Delgado E, Hak E, Hoepelman IM. Application of guidelines on preoperative antibiotic prophylaxis in Leon, Nicaragua. Neth J Med [Internet]. 2006;64(11):411–6. Available from: https://www.ncbi.nlm.nih.gov/pubmed/17179571 17179571

[13] Vessal G, Namazi S, Davarpanah MA, Foroughinia F. Evaluation of prophylactic antibiotic administration at the surgical ward of a major referral hospital, Islamic Republic of Iran. East Mediterr Heal J. 2011;17:663–8. 21977569

[14] Elbur AI, Yousif MAER, Elsayed ASA, Abdel-Rahman ME. An audit of prophylactic surgical antibiotic use in a Sudanese Teaching Hospital. Int J Clin Pharm. 2013;35(1):149–53. 10.1007/s11096-012-9719-y 23135836

[15] Abdel-Aziz A, El-Menyar A, Al-Thani H, Zarour A, Parchani A, Asim M, et al Adherence of surgeons to antimicrobial prophylaxis guidelines in a tertiary general hospital in a rapidly developing country. Adv Pharmacol Sci. 2013;2013. 10.1155/2013/842593 PMC3885161 24454349

[16] Al-azzam SI, Alzoubi KH, Mhaidat NM, Haddadin RD, Masadeh MM, Tumah HN, et al Preoperative antibiotic prophylaxis practice and guideline adherence in Jordan: a multi-centre study in Jordani. J Infect Dev Ctries. 2012;6(10):715–20. 10.3855/jidc.1676 23103893

[17] Oh AL, Goh LM, Nik Azim NA, Tee CS, Phung Shehab CW. Antibiotic usage in surgical prophylaxis: A prospective surveillance of surgical wards at a tertiary hospital in Malaysia. J Infect Dev Ctries. 2014;8(2):193–201. 10.3855/jidc.3076 24518629

[18] Parulekar L, Soman R, Singhal T, Rodrigues C, Dastur FD, Mehta A. How good is compliance with surgical antibiotic prophylaxis guidelines in a tertiary care private hospital in India? A prospective study. Indian J Surg. 2009;71:15–8. 10.1007/s12262-009-0004-9 23133102 PMC3452567

