# Supplementary material for: Antibiotic usage in surgical prophylaxis: A prospective observational study in the surgical ward of Nekemte referral hospital
Source: PLoS One. 2018 Sep 13;13(9):e0203523. doi: 10.1371/journal.pone.0203523 (PMC6136737; doi:10.1371/journal.pone.0203523)
Supplement: S1 Table — (DOCX) [file pone.0203523.s001.docx]

Table 1: Characteristics of surgical inpatients taking SAP in NRH from 1^st^ April to 30^th^ June, 2017

| **Variable** | **Frequency (N=153) (%)** |
| --- | --- |
| **Age** (Median (IQR)) | 35.0 (25-50) |
| **Sex** |  |
| Male | 90 (58.8) |
| Female | 63 (41.2) |
| **LoS** | 8.0 (5-11) |
| **Final status** |  |
| Improved | 149 (97.4) |
| Dead | 4 (2.6) |
| **Ward** |  |
| General surgical | 92 (60.1) |
| Gynecology-obstetrics | 38 (24.8) |
| Orthopedic | 23 (15.0) |

## SAP providers’ demographics

Except one, all of the study participants receive a prophylaxis provided by nurses. The majority of them (76.5%) were females. Above half of the providers were in the age range of 30-40 years and most of them have an experience of 8-10 years **(Table 2)**.

Table 2: Socio-demographic characteristics of the surgical prophylaxis providers in NRH from 1^st^ April to 30^th^ June 2017

| **Age of provider** | **Frequency (n=153) (%)** |
| --- | --- |
| < 30 years | 45 (29.4) |
| 30-40 years | 81 (52.9) |
| > 40 years | 27 (17.6) |
| **Sex of provider** |  |
| Male | 36 (23.5) |
| Female | 117 (76.5) |
| **Experience of provider** |  |
| 8-10 years | 91 (59.5) |
| > 10 Years | 62 (40.5) |

## Perioperative characteristics of surgical patients

Most of the surgical cases were gastrointestinal (39.2%) followed by gynecology and obstetrics (15.7%). Majority of the procedures were clean (40.7%), followed by clean-contaminated (32%). Thirteen (8.5%) patients had a urinary catheter. Most of the surgeries took 1-2 hours (56.2%) and about 67% of them were performed in the morning. Sixty-eight (44.4%) of the patients took 2-5 days course of antibiotics. Less than a quarter (20.9%) of the patients took surgical antibiotics for not greater than a day. About 80 % of the antibiotics were started before surgical incision. Above fifty percent of the patients took the surgical antibiotics preoperatively, within one hour of surgical incision **(Table 3)**.

Table 3: Preoperative characteristics of study participants at NRH from 1^st^ April to 30^th^ June, 2017

| **Perioperative characteristics** | **Frequency (%)** |
| --- | --- |
| **Class of Surgery** |  |
| Gastrointestinal | 60 (39.2) |
| Gynecology and obstetrics | 38 (24.8) |
| Orthopedic | 24 (15.7) |
| Urologic | 16 (10.5) |
| Head and neck | 3 (2.0) |
| Others | 12 (7.8) |
| **Wound Class (n=153)** |  |
| Clean | 66 (43.1) |
| Clean-contaminated | 49 (32.0) |
| Contaminated | 38 (24.8) |
| **Presence of Catheter** |  |
| Yes (Catheter (13) | 13 (8.5) |
| No | 140 (91.5) |
| **Duration of surgery (hours) (n=**153**)** |  |
| < 1 | 66 (43.1) |
| 1-2 | 86 (56.2) |
| > 2 | 1 (0.7) |
| **Shift surgery done** |  |
| Morning | 103 (67.3) |
| After | 20 (13.1) |
| Before mid-night | 26 (17.0), |
| After mid-night | 4 (2.6) |
| **Duration of prophylaxis administration (days)** |  |
| One day | 32 (20.9) |
| 2-5 days | 68 (44.4) |
| 6-7 days | 34 (22.2) |
| 8-14 days | 15 (9.8) |
| >15 days | 4 (2.6) |
| **Timing of prophylaxis** |  |
| Before incision | 122 (79.7) |
| After incision | 31 (20.3) |
| **Timing compliance with the intervals (n=153)** |  |
| Early | 42 (27.5) |
| Preoperation | 80 (52.3) |
| Perioperative | 20 (13.1) |
| Postoperative | 11 (7.2) |

## Surgical type and procedures

Sixty (39.2%) surgical cases were gastrointestinal. Appendicitis is the most common GI diagnosis (19/60), followed by colorectal cases (17/60) making appendectomy the most frequently performed gastrointestinal procedure. Among the 38 gynecology and obstetrics case, 33 were gynecologic only (17 Utero-vaginal prolapses). Out of 24 orthopedic procedures, 23 were different types of fractures. Eight external fixations and seven open reduction and internal fixations (ORIF) were the most commonly performed orthopedic procedures. Among the 16 urologic procedures, 12 were Prostatectomy followed by 3 hydrocelectomy procedures **(Table 4)**.

Table 4: Surgical type and procedures of surgical inpatients at NRH from 1^st^ April to 30^th^ June 2017

| **Surgery type (F)** | **Diagnosis (F)** | **Procedure (F)** | **F (%)** |
| --- | --- | --- | --- |
| **GI** |  |  | **60 (39.2)** |
| Gastro-duodenal/ General (11) | Perforated abdomen (4), | Repair with Graham’s Patch (4) |  |
|  | Penetrating abdomen (2), blunt abdominal trauma (2) | Laparotomy (4) |  |
|  | Mesenteric cyst (1) | Excision (1) |  |
|  | Gastric outlet obstruction (1), Post-operative adhesion (1) | Gastrojejunostomy (1), Repair (1) |  |
| Biliary Tract (3) | Cholelithiasis (2), gallbladder stone (1) | Cholecystectomy (2), Laparotomy (1) |  |
| Appendectomy (19) | Appendicitis (19) | Appendectomy (19) |  |
| Small bowel (7) | Small bowel obstruction (6), | R+A (6), |  |
| Hernia (4) | Hernia (4) | Herniorrhaphy (4) |  |
| Colorectal (17) | Large bowel obstruction (8), | R+A (6), laparotomy (2), |  |
|  | Colostomy (4), | Colostomy closure (2), Permanent colostomy (2) |  |
|  | Rectal Cancer (2), hemorrhoid (2), Perianal fistula (1) | Permanent colostomy (1), Hemorrhoidectomy (2), Fistulectomy (1) |  |
| **Gynecology and obstetric** | Utero-vaginal prolapse (17) | Vaginal Hysterectomy (17) | **38 (24.8)** |
|  | Myoma (9), Endometrial cancer (1) | Myomectomy (8), Total Abdominal Hysterectomy (2) |  |
|  | Adnexal Cyst/Tumor (6), | Salpingectomy (4), laparotomy (1), Cystectomy (1) |  |
|  | Antepartum hemorrhage /Uterine Rupture(5) | Laparotomy (2), TAH (2), Bilateral tubal ligation (1) |  |
| **Orthopedic** **surgery** | Fracture (23) | Debridement (3), External Fixation (8), Gator (3), ORIF (6), TBW (3) | **24 (15.7)** |
|  | Gangrene (1) | Amputation |  |
| **Urologic surgery** |  |  | **16 (10.5)** |
|  | BPH (12) | Prostatectomy (12) |  |
|  | Hydrocele (3) | Hydrocelecectomy (3) |  |
|  | Hydronephrosis (1) | R+A (1) |  |
| **Head and neck** |  |  | **3 (2.0)** |
|  | Goiter (3) | Thyroidectomy (3) |  |
| **Others surgeries** |  |  | **12 (7.8)** |
| - Skin and deep tissue (5) | Skin cancer (1)/ Lipoma (1) | Excision (2) |  |
|  | Fasciitis (1)/ Malunion (1)/Soft tissue injury (1) | Fasciectomy (1), skin graft (1), repair (1) |  |
| - Breast (2) | Breast mass/cancer | Mastectomy (2), |  |
| - Miscellaneous | Pelvic mass (1), Popliteal cysts (1), | Excision (2) |  |
|  | wound dehiscence (1) | Wound Closure |  |
|  | Stab injury (1), animal bite (1) | Laparotomy (2) |  |
| **Total** |  |  | **153 (100)** |

*F: frequency, GI: gastrointestinal, TAH: Total Abdominal Hysterectomy, R & A: resection and anastomosis, ORIF: open reduction and internal fixations, TBW: tension banding and wiring,*

## Antibiotics used for prophylaxis

About 59% of patients took a single prophylactic drug and about 39 % took a combination of two drugs. Namely, about 84 % of the participants received Ceftriaxone. Metronidazole (35.3 %) is the second most prescribed prophylactic antimicrobial followed by ampicillin (19.6 %) **(Table 5)**.

Table 5: Utilization pattern of SAP among surgical patients at NRH from 1^st^ April to 30^th^ June 2017

| **Variables** | **Frequency (n=153) (%)** |
| --- | --- |
| **Number of prophylactic antibiotic(s) used (n=153)** |  |
| One | 90 (58.8) |
| Two | 60 (39.2) |
| Three | 3 (2.0 |
| **Name of Prophylactic antibiotics used** |  |
| Amoxicillin | 1 (0.7) |
| Ampicillin | 21 (13.7) |
| Cloxacillin | 2 (1.3) |
| Ceftriaxone | 66 (43.1) |
| Ceftriaxone + Ampicillin | 6 (3.9) |
| Ceftriaxone + Ampicillin + Metronidazole | 3 (2.0) |
| Ceftriaxone + Metronidazole | 51 (33.3) |
| Ceftriaxone + Cloxacillin | 3 (2.0) |
| **Specific prophylactic antibiotic used per patient** |  |
| Ceftriaxone | 129 (84.3) |
| Ampicillin | 54 (35.3) |
| Metronidazole | 30 (19.6) |
| Cloxacillin | 5 (3.3) |
| Amoxicillin | 1 (0.7) |
| **Prophylactic antibiotic use duration (n=153)** |  |
| Median (interquartile range) (days) | 5.0 (3-7) |

## SAP compliance to the guidelines

About 20 % of the prophylactic drugs were given for cases that lack specific recommendation as per the ASHP guideline. Among the reaming (80.4%) recommended indications, only 10.6 % of the selections were adequate/compliant with the guideline recommendation. Majority of the (67.5%) selections were unrelated to the recommendations and 19.5% were unnecessarily border than the guideline recommendations. On the other hand, surprisingly all of the administrations were non-concordant to the Ethiopian national STG for general hospitals **(Table 6)**.

Table 6: SAP use and selection compliance to national STG and ASHP guidelines among surgical patients in NRH from 1^st^ April to 30^th^ June 2017

| **Prophylaxis Indication (n=153)** | **STG** | | **ASHP** | |
| --- | --- | --- | --- | --- |
| **Indication compliance** | **Frequency** | **%** | **Frequency** | **%** |
| Given with Indication | 126 | 82.4 | 123 | 80.4 |
| Given without indication | 27 | 17.6 | 30 | 19.6 |
| **Selection compliance** | **(n=126)** |  | **(n=123)** |  |
| Adequate/concordant | 0 | 0 | 13 | 10.6 |
| Narrow | 0 | 0 | 3 | 2.4 |
| Broader | 42 | 33.3 | 24 | 19.5 |
| Unrelated | 84 | 66.7 | 83 | 67.5 |

## SAP duration and preoperative timing

Majority of the prophylactic antibiotics were administered for greater than 24 hours (75.8%) and about 48 % of the administrations were not given in the appropriate preoperative period of within 0 to 60 minutes before incision as recommended by the guidelines (52.3) **(Table 7)**.

Table 7: Duration and timing of SAP among surgical inpatients in NRH from 1^st^ April to 30^th^ June 2017

| **Variable** | **Frequency (n=153)** | **%** |
| --- | --- | --- |
| Duration less or equal to 24 hours |  |  |
| Yes | 37 | 24.2 |
| No | 116 | 75.8 |
| Timing within 0 to 60 minutes prior to incision |  |  |
| Yes | 80 | 52.3 |
| No | 73 | 47.7 |

## Factors affecting Timing and duration of SAP

### Factors affecting the timing of SAP administration

Table 8: The univariate analysis of the factors affecting timing of SAP among surgical inpatients in NRH from 1^st^ April to 30^th^ June 2017

| **Variables** | **Timing (not within 60 minutes before incision)** | **COR (95% C.I.)** | **Sig.** |
| --- | --- | --- | --- |
| Sex (Male) | 55 (61.1) | 3.93 (1.97, 7.85) | 0.000 |
| Age |  | 1.01 (0.99, 1.03) | 0.563 |
| Ward |  |  |  |
| Surgical | 52 (56.5) | 1.00 (0.40, 2.51) | 1.000 |
| Genecology and obstetric | 8 (21.1) | 0.21 (0.07, 0.64) | 0.006 |
| Orthopedic | 13(56.5) | [Reference] |  |
| Surgery type (Emergent) | 44 (57.9) | 2.28 (1.19, 4.35) | 0.013 |
| Wound class |  |  |  |
| Clean | 35(53.0) | 0.74 (0.33, 1.66) | 0.459 |
| Clean-contaminated | 15 (30.6) | 0.29 (0.12, 0.70) | 0.006 |
| Contaminated | 23 (60.5) | [Reference] |  |
| Duration of surgery |  | 0.99 (0.99, 1.01) | 0.703 |
| Presence of medical device |  | 1.64 (0.52, 5.14) | 0.399 |
| 24 hour and less SAP duration | 10 (27) | 0.31 (0.14, 0.70) | 0.005 |
| Sex of the provider (Male) | 27 (75.0) | 4.63 (1.99, 10.73) | 0.000 |
| Age of the provider |  |  |  |
| Age (<3o years) | 12 (26.7) | 0.83 (0.026, 0.27) | 0.000 |
| Age (30-40 years) | 39 (48.1) | 0.21 (0.07, 0.61) | 0.004 |
| > 40 years | 22 (81.5) | [Reference] |  |
| Experience of provider (< 10 years) | 35 (38.5) | 0.40 (0.20, 0.77) | 0.006 |

Emergent surgery procedures (AOR= 2.89, 95% CI: (1.09-9.10) and being a male patient (AOR=3.10, 95% CI: 1.07-8.98) were the two factors that were associated with the inappropriate timing of a prophylactic administration as per the guidelines **(Table 8)**.

Table 9: The multivariate analysis of the factors that determine the timing of SAP administration among surgical patients in NRH from 1^st^ April to 30^th^ June, 2017

| **Variables** | **Timing (not within 60 minutes before incision)** |  |  | **AOR (95% C.I.)** | **Sig.** |
| --- | --- | --- | --- | --- | --- |
| Sex (Male) | 55 (61.1) |  |  | 3.10 (1.07, 8.98) | 0.037 |
| Ward |  |  |  |  |  |
| Surgical | 52 (56.5) |  |  | 1.11(0.32, 3.83) | 0.873 |
| Genecology and obstetric | 8 (21.1) |  |  | 0.76 (0.06, 8.79)) | 0.823 |
| Orthopedic | 13(56.5) |  |  | [Reference] |  |
| Surgery type (Emergent) | 44 (57.9) |  |  | 2.89 (1.09, 9.10) | 0.049 |
| Wound class |  |  |  |  |  |
| Clean | 35(53.0) |  |  | 1.66 (0.54, 5.08) | 0.376 |
| Clean-contaminated | 15 (30.6) |  |  | 0.69 (0.21, 2.23) | 0.525 |
| Contaminated | 23 (60.5) |  |  | [Reference] |  |
| Greater than 24hr SAP duration | 10 (27) |  |  | 0.55 (0.18, 1.69) | 0.292 |
| Sex of the provider (Male) | 27 (75.0) |  |  | 1.52 (0.36, 6.30) | 0.568 |
| Age of the provider |  |  |  |  |  |
| Age (<3o years) | 12 (26.7) |  |  | 0.31 (0.03, 3.03) | 0.312 |
| Age (30-40 years) | 39 (48.1) |  |  | 0.17 (0.02, 1.220 | 0.078 |
| > 40 years | 22 (81.5) |  |  | [Reference] |  |
| Experience of provider (< 10 years) | 35 (38.5) |  |  | 1.40 (0.53, 3.67) | 0.494 |

### Factors associated with greater than 24 hour SAP use duration

Table 10: The univariate analysis of the factors attributing for long duration of SAP use among surgical inpatients in NRH from 1^st^ April to 30^th^ June 2017

| **Variables** | **> 24 hour SAP duration, frequency (%)** | **COR (95% C.I.)** | **Sig.** |
| --- | --- | --- | --- |
| Age |  | 1.00 (0.98, 1.02) | 0.991 |
| Sex (Male) | 82 (91.1) | 8.74 (3.63, 21.06) | 0.001 |
| Surgery type (emergent) | 66 (86.8) | 3.56 (1.58, 8.04) | 0.002 |
| Ward |  |  |  |
| Surgical | 86 (93.5) | 3.02 (0.78, 11.75) | 0.11 |
| Genecology & obstetric | 11 (28.9) | 0.09 (0.02, 0.31) | 0.000 |
| Orthopedic | 19 (82.6) | [Reference] |  |
| Duration of surgery |  | 0.99 (0.98, 1.01) | .746 |
| Length of stay (< 8 days) | 63 (66.3) | 0.19 (0.07, 0.51) | 0.001 |
| Sex of Provider(Male) | 32 (88.9) | 3.14 (1.03, 9.58) | 0.044 |
| Experience (< 10 years) | 61 (67.0) | 0.26 (0.11, 0.64) | 0.003 |
| Age of provider |  |  |  |
| < 30 years | 18 (45) | 0.12 (0.13, 1.39) | 0.231 |
| 30-40 years | 75 (81) | 2.17 (0.56, 8.37) | 0.259 |
| > 40 years | 23 (27) | [Reference] |  |

The only ward of admission has an independent association with SAP use duration. Patients admitted to Gynecology and obstetrics ward were more likely to receive SAP for less than or equal to 24 hour days (AOR=0.07, 95% CI: 0.01-0.81) than patients admitted to orthopedic ward **(Table 9)**.

Table 11: The multivariate analysis of the factors associated with prolonged SAP use duration among surgical patients in NRH from 1^st^ April to 30^th^ June 2017

| **Variables** | **> 24 hour SAP use duration** |  |  | **AOR (95% C.I.)** | **Sig.** |
| --- | --- | --- | --- | --- | --- |
| Sex (Male) | 82 (91.1) |  |  | 1.01 (0.18, 5.52) | 0.994 |
| Surgery type (emergent) | 66 (86.8) |  |  | 1.14 (0.33, 3.94) | 0.839 |
| Ward |  |  |  |  |  |
| Surgical | 86 (93.5) |  |  | 2.82 (0.62, 12.81) | 0.178 |
| Genecology & obstetric | 11 (28.9) |  |  | 0.07 (0.01, 0.81) | 0.033 |
| Orthopedic | 19 (82.6) |  |  | [Reference] |  |
| Sex of Provider(Male) | 32 (88.9) |  |  | 1.18 (0.29, 4.88) | 0.817 |
| Length of stay (< 8 days) | 63 (66.3) |  |  | 0.78 (0.22, 2.80) | 0.702 |
| Experience (< 10 years) | 61 (67.0) |  |  | 1.94 (0.44, 8.50) | 0.378 |
