# Supplementary material for: Antibiotic usage in surgical prophylaxis: A prospective observational study in the surgical ward of Nekemte referral hospital
Source: PLoS One. 2018 Sep 13;13(9):e0203523. doi: 10.1371/journal.pone.0203523 (PMC6136737; doi:10.1371/journal.pone.0203523)
Supplement: S2 Table — (DOCX) [file pone.0203523.s002.docx]

Table 2: Socio-demographic characteristics of the surgical prophylaxis providers in NRH from 1^st^ April to 30^th^ June 2017

| **Age of provider** | **Frequency (n=153) (%)** |
| --- | --- |
| < 30 years | 45 (29.4) |
| 30-40 years | 81 (52.9) |
| > 40 years | 27 (17.6) |
| **Sex of provider** |  |
| Male | 36 (23.5) |
| Female | 117 (76.5) |
| **Experience of provider** |  |
| 8-10 years | 91 (59.5) |
| > 10 Years | 62 (40.5) |
