# Supplementary material for: Antibiotic usage in surgical prophylaxis: A prospective observational study in the surgical ward of Nekemte referral hospital
Source: PLoS One. 2018 Sep 13;13(9):e0203523. doi: 10.1371/journal.pone.0203523 (PMC6136737; doi:10.1371/journal.pone.0203523)
Supplement: S3 Table — (DOCX) [file pone.0203523.s003.docx]

Table 3: Preoperative characteristics of study participants at NRH from 1^st^ April to 30^th^ June, 2017

| **Perioperative characteristics** | **Frequency (%)** |
| --- | --- |
| **Class of Surgery** |  |
| Gastrointestinal | 60 (39.2) |
| Gynecology and obstetrics | 38 (24.8) |
| Orthopedic | 24 (15.7) |
| Urologic | 16 (10.5) |
| Head and neck | 3 (2.0) |
| Others | 12 (7.8) |
| **Wound Class (n=153)** |  |
| Clean | 66 (43.1) |
| Clean-contaminated | 49 (32.0) |
| Contaminated | 38 (24.8) |
| **Presence of Catheter** |  |
| Yes (Catheter (13) | 13 (8.5) |
| No | 140 (91.5) |
| **Duration of surgery (hours) (n=**153**)** |  |
| < 1 | 66 (43.1) |
| 1-2 | 86 (56.2) |
| > 2 | 1 (0.7) |
| **Shift surgery done** |  |
| Morning | 103 (67.3) |
| After | 20 (13.1) |
| Before mid-night | 26 (17.0), |
| After mid-night | 4 (2.6) |
| **Duration of prophylaxis administration (days)** |  |
| One day | 32 (20.9) |
| 2-5 days | 68 (44.4) |
| 6-7 days | 34 (22.2) |
| 8-14 days | 15 (9.8) |
| >15 days | 4 (2.6) |
| **Timing of prophylaxis** |  |
| Before incision | 122 (79.7) |
| After incision | 31 (20.3) |
| **Timing compliance with the intervals (n=153)** |  |
| Early | 42 (27.5) |
| Preoperation | 80 (52.3) |
| Perioperative | 20 (13.1) |
| Postoperative | 11 (7.2) |
