# Supplementary material for: Antibiotic usage in surgical prophylaxis: A prospective observational study in the surgical ward of Nekemte referral hospital
Source: PLoS One. 2018 Sep 13;13(9):e0203523. doi: 10.1371/journal.pone.0203523 (PMC6136737; doi:10.1371/journal.pone.0203523)
Supplement: S4 Table — F: frequency, TAH: Total Abdominal Hysterectomy, R & A: resection and anastomosis, ORIF: open reduction and internal fixations, TBW: tension banding and wiring. (DOCX) [file pone.0203523.s004.docx]

Table 4: Surgical type and procedures of surgical inpatients at NRH from 1^st^ April to 30^th^ June 2017

| **Surgery type (F)** | **Diagnosis (F)** | **Procedure (F)** | **F (%)** |
| --- | --- | --- | --- |
| **GI** |  |  | **60 (39.2)** |
| Gastro-duodenal/ General (11) | Perforated abdomen (4), | Repair with Graham’s Patch (4) |  |
|  | Penetrating abdomen (2), blunt abdominal trauma (2) | Laparotomy (4) |  |
|  | Mesenteric cyst (1) | Excision (1) |  |
|  | Gastric outlet obstruction (1), Post-operative adhesion (1) | Gastrojejunostomy (1), Repair (1) |  |
| Biliary Tract (3) | Cholelithiasis (2), gallbladder stone (1) | Cholecystectomy (2), Laparotomy (1) |  |
| Appendectomy (19) | Appendicitis (19) | Appendectomy (19) |  |
| Small bowel (7) | Small bowel obstruction (6), | R+A (6), |  |
| Hernia (4) | Hernia (4) | Herniorrhaphy (4) |  |
| Colorectal (17) | Large bowel obstruction (8), | R+A (6), laparotomy (2), |  |
|  | Colostomy (4), | Colostomy closure (2), Permanent colostomy (2) |  |
|  | Rectal Cancer (2), hemorrhoid (2), Perianal fistula (1) | Permanent colostomy (1), Hemorrhoidectomy (2), Fistulectomy (1) |  |
| **Gynecology and obstetric** | Utero-vaginal prolapse (17) | Vaginal Hysterectomy (17) | **38 (24.8)** |
|  | Myoma (9), Endometrial cancer (1) | Myomectomy (8), Total Abdominal Hysterectomy (2) |  |
|  | Adnexal Cyst/Tumor (6), | Salpingectomy (4), laparotomy (1), Cystectomy (1) |  |
|  | Antepartum hemorrhage /Uterine Rupture(5) | Laparotomy (2), TAH (2), Bilateral tubal ligation (1) |  |
| **Orthopedic** **surgery** | Fracture (23) | Debridement (3), External Fixation (8), Gator (3), ORIF (6), TBW (3) | **24 (15.7)** |
|  | Gangrene (1) | Amputation |  |
| **Urologic surgery** |  |  | **16 (10.5)** |
|  | BPH (12) | Prostatectomy (12) |  |
|  | Hydrocele (3) | Hydrocelecectomy (3) |  |
|  | Hydronephrosis (1) | R+A (1) |  |
| **Head and neck** |  |  | **3 (2.0)** |
|  | Goiter (3) | Thyroidectomy (3) |  |
| **Others surgeries** |  |  | **12 (7.8)** |
| - Skin and deep tissue (5) | Skin cancer (1)/ Lipoma (1) | Excision (2) |  |
|  | Fasciitis (1)/ Malunion (1)/Soft tissue injury (1) | Fasciectomy (1), skin graft (1), repair (1) |  |
| - Breast (2) | Breast mass/cancer | Mastectomy (2), |  |
| - Miscellaneous | Pelvic mass (1), Popliteal cysts (1), | Excision (2) |  |
|  | wound dehiscence (1) | Wound Closure |  |
|  | Stab injury (1), animal bite (1) | Laparotomy (2) |  |
| **Total** |  |  | **153 (100)** |

*F: frequency, GI: gastrointestinal, TAH: Total Abdominal Hysterectomy, R & A: resection and anastomosis, ORIF: open reduction and internal fixations, TBW: tension banding and wiring,*
