# Supplementary material for: Antibiotic usage in surgical prophylaxis: A prospective observational study in the surgical ward of Nekemte referral hospital
Source: PLoS One. 2018 Sep 13;13(9):e0203523. doi: 10.1371/journal.pone.0203523 (PMC6136737; doi:10.1371/journal.pone.0203523)
Supplement: S5 Table — (DOCX) [file pone.0203523.s005.docx]

Table 5: Utilization pattern of SAP among surgical patients at NRH from 1^st^ April to 30^th^ June 2017

| **Variables** | **Frequency (n=153) (%)** |
| --- | --- |
| **Number of prophylactic antibiotic(s) used (n=153)** |  |
| One | 90 (58.8) |
| Two | 60 (39.2) |
| Three | 3 (2.0 |
| **Name of Prophylactic antibiotics used** |  |
| Amoxicillin | 1 (0.7) |
| Ampicillin | 21 (13.7) |
| Cloxacillin | 2 (1.3) |
| Ceftriaxone | 66 (43.1) |
| Ceftriaxone + Ampicillin | 6 (3.9) |
| Ceftriaxone + Ampicillin + Metronidazole | 3 (2.0) |
| Ceftriaxone + Metronidazole | 51 (33.3) |
| Ceftriaxone + Cloxacillin | 3 (2.0) |
| **Specific prophylactic antibiotic used per patient** |  |
| Ceftriaxone | 129 (84.3) |
| Ampicillin | 54 (35.3) |
| Metronidazole | 30 (19.6) |
| Cloxacillin | 5 (3.3) |
| Amoxicillin | 1 (0.7) |
| **Prophylactic antibiotic use duration (n=153)** |  |
| Median (interquartile range) (days) | 5.0 (3-7) |
