# Supplementary material for: Antibiotic usage in surgical prophylaxis: A prospective observational study in the surgical ward of Nekemte referral hospital
Source: PLoS One. 2018 Sep 13;13(9):e0203523. doi: 10.1371/journal.pone.0203523 (PMC6136737; doi:10.1371/journal.pone.0203523)
Supplement: S6 Table — (DOCX) [file pone.0203523.s006.docx]

Table 6: SAP compliance to national STG and ASHP guidelines among surgical patients in NRH from 1^st^ April to 30^th^ June 2017

| **Prophylaxis Indication (n=153)** | **STG** | | **ASHP** | |
| --- | --- | --- | --- | --- |
| **Indication compliance** | **Frequency** | **%** | **Frequency** | **%** |
| Given with Indication | 126 | 82.4 | 123 | 80.4 |
| Given without indication | 27 | 17.6 | 30 | 19.6 |
| **Selection compliance** | **(n=126)** |  | **(n=123)** |  |
| Adequate/concordant | 0 | 0 | 13 | 10.6 |
| Narrow | 0 | 0 | 3 | 2.4 |
| Broader | 42 | 33.3 | 24 | 19.5 |
| Unrelated | 84 | 66.7 | 83 | 67.5 |
