# Supplementary material for: Antibiotic usage in surgical prophylaxis: A prospective observational study in the surgical ward of Nekemte referral hospital
Source: PLoS One. 2018 Sep 13;13(9):e0203523. doi: 10.1371/journal.pone.0203523 (PMC6136737; doi:10.1371/journal.pone.0203523)
Supplement: S7 Table — (DOCX) [file pone.0203523.s007.docx]

Table 7: Duration and timing of SAP among surgical inpatients in NRH from 1^st^ April to 30^th^ June 2017

| **Variable** | **Frequency (n=153)** | **%** |
| --- | --- | --- |
| Duration less or equal to 24 hours |  |  |
| Yes | 37 | 24.2 |
| No | 116 | 75.8 |
| Timing within 0 to 60 minutes prior to incision |  |  |
| Yes | 80 | 52.3 |
| No | 73 | 47.7 |
