# Supplementary material for: Antibiotic usage in surgical prophylaxis: A prospective observational study in the surgical ward of Nekemte referral hospital
Source: PLoS One. 2018 Sep 13;13(9):e0203523. doi: 10.1371/journal.pone.0203523 (PMC6136737; doi:10.1371/journal.pone.0203523)
Supplement: S8 Table — (DOCX) [file pone.0203523.s008.docx]

Table 8. The univariate analysis of the factors affecting the timing of SAP among surgical inpatients in NRH from 1st April to 30th June 2017

| **Variables** | **Timing (not within 60 minutes before incision)** | **COR (95% C.I.)** | **Sig.** |
| --- | --- | --- | --- |
| Sex (Male) | 55 (61.1) | 3.93 (1.97, 7.85) | 0.000 |
| Age |  | 1.01 (0.99, 1.03) | 0.563 |
| Ward |  |  |  |
| Surgical | 52 (56.5) | 1.00 (0.40, 2.51) | 1.000 |
| Genecology and obstetric | 8 (21.1) | 0.21 (0.07, 0.64) | 0.006 |
| Orthopedic | 13(56.5) | [Reference] |  |
| Surgery type (Emergent) | 44 (57.9) | 2.28 (1.19, 4.35) | 0.013 |
| Wound class |  |  |  |
| Clean | 35(53.0) | 0.74 (0.33, 1.66) | 0.459 |
| Clean-contaminated | 15 (30.6) | 0.29 (0.12, 0.70) | 0.006 |
| Contaminated | 23 (60.5) | [Reference] |  |
| Duration of surgery |  | 0.99 (0.99, 1.01) | 0.703 |
| Presence of medical device |  | 1.64 (0.52, 5.14) | 0.399 |
| 24 hour and less SAP duration | 10 (27) | 0.31 (0.14, 0.70) | 0.005 |
| Sex of the provider (Male) | 27 (75.0) | 4.63 (1.99, 10.73) | 0.000 |
| Age of the provider |  |  |  |
| Age (<3o years) | 12 (26.7) | 0.83 (0.026, 0.27) | 0.000 |
| Age (30-40 years) | 39 (48.1) | 0.21 (0.07, 0.61) | 0.004 |
| > 40 years | 22 (81.5) | [Reference] |  |
| Experience of provider (< 10 years) | 35 (38.5) | 0.40 (0.20, 0.77) | 0.006 |
