# Supplementary material for: Antibiotic usage in surgical prophylaxis: A prospective observational study in the surgical ward of Nekemte referral hospital
Source: PLoS One. 2018 Sep 13;13(9):e0203523. doi: 10.1371/journal.pone.0203523 (PMC6136737; doi:10.1371/journal.pone.0203523)
Supplement: S9 Table — (DOCX) [file pone.0203523.s009.docx]

Table 9: The multivariate analysis of the factors that determine the timing of SAP administration among surgical patients in NRH from 1^st^ April to 30^th^ June, 2017

| **Variables** | **Timing (not within 60 minutes before incision)** |  |  | **AOR (95% C.I.)** | **Sig.** |
| --- | --- | --- | --- | --- | --- |
| Sex (Male) | 55 (61.1) |  |  | 3.10 (1.07, 8.98) | 0.037 |
| Ward |  |  |  |  |  |
| Surgical | 52 (56.5) |  |  | 1.11(0.32, 3.83) | 0.873 |
| Genecology and obstetric | 8 (21.1) |  |  | 0.76 (0.06, 8.79)) | 0.823 |
| Orthopedic | 13(56.5) |  |  | [Reference] |  |
| Surgery type (Emergent) | 44 (57.9) |  |  | 2.89 (1.09, 9.10) | 0.049 |
| Wound class |  |  |  |  |  |
| Clean | 35(53.0) |  |  | 1.66 (0.54, 5.08) | 0.376 |
| Clean-contaminated | 15 (30.6) |  |  | 0.69 (0.21, 2.23) | 0.525 |
| Contaminated | 23 (60.5) |  |  | [Reference] |  |
| Greater than 24hr SAP duration | 10 (27) |  |  | 0.55 (0.18, 1.69) | 0.292 |
| Sex of the provider (Male) | 27 (75.0) |  |  | 1.52 (0.36, 6.30) | 0.568 |
| Age of the provider |  |  |  |  |  |
| Age (<3o years) | 12 (26.7) |  |  | 0.31 (0.03, 3.03) | 0.312 |
| Age (30-40 years) | 39 (48.1) |  |  | 0.17 (0.02, 1.220 | 0.078 |
| > 40 years | 22 (81.5) |  |  | [Reference] |  |
| Experience of provider (< 10 years) | 35 (38.5) |  |  | 1.40 (0.53, 3.67) | 0.494 |
