# Supplementary material for: Antibiotic usage in surgical prophylaxis: A prospective observational study in the surgical ward of Nekemte referral hospital
Source: PLoS One. 2018 Sep 13;13(9):e0203523. doi: 10.1371/journal.pone.0203523 (PMC6136737; doi:10.1371/journal.pone.0203523)
Supplement: S10 Table — (DOCX) [file pone.0203523.s010.docx]

Table 10. The univariate analysis of the factors attributing for long duration of SAP use among surgical inpatients in NRH from 1^st^ April to 30^th^ June 2017

| **Variables** | **> 24 hour SAP duration, frequency (%)** | **COR (95% C.I.)** | **Sig.** |
| --- | --- | --- | --- |
| Age |  | 1.00 (0.98, 1.02) | 0.991 |
| Sex (Male) | 82 (91.1) | 8.74 (3.63, 21.06) | 0.001 |
| Surgery type (emergent) | 66 (86.8) | 3.56 (1.58, 8.04) | 0.002 |
| Ward |  |  |  |
| Surgical | 86 (93.5) | 3.02 (0.78, 11.75) | 0.11 |
| Genecology & obstetric | 11 (28.9) | 0.09 (0.02, 0.31) | 0.000 |
| Orthopedic | 19 (82.6) | [Reference] |  |
| Duration of surgery |  | 0.99 (0.98, 1.01) | .746 |
| Length of stay (< 8 days) | 63 (66.3) | 0.19 (0.07, 0.51) | 0.001 |
| Sex of Provider(Male) | 32 (88.9) | 3.14 (1.03, 9.58) | 0.044 |
| Experience (< 10 years) | 61 (67.0) | 0.26 (0.11, 0.64) | 0.003 |
| Age of provider |  |  |  |
| < 30 years | 18 (45) | 0.12 (0.13, 1.39) | 0.231 |
| 30-40 years | 75 (81) | 2.17 (0.56, 8.37) | 0.259 |
| > 40 years | 23 (27) | [Reference] |  |
