# Supplementary material for: Antibiotic usage in surgical prophylaxis: A prospective observational study in the surgical ward of Nekemte referral hospital
Source: PLoS One. 2018 Sep 13;13(9):e0203523. doi: 10.1371/journal.pone.0203523 (PMC6136737; doi:10.1371/journal.pone.0203523)
Supplement: S11 Table — (DOCX) [file pone.0203523.s011.docx]

Table 11: The multivariate analysis of the factors associated with prolonged SAP use duration among surgical patients in NRH from 1^st^ April to 30^th^ June 2017

| **Variables** | **> 24 hour SAP use duration** |  |  | **AOR (95% C.I.)** | **Sig.** |
| --- | --- | --- | --- | --- | --- |
| Sex (Male) | 82 (91.1) |  |  | 1.01 (0.18, 5.52) | 0.994 |
| Surgery type (emergent) | 66 (86.8) |  |  | 1.14 (0.33, 3.94) | 0.839 |
| Ward |  |  |  |  |  |
| Surgical | 86 (93.5) |  |  | 2.82 (0.62, 12.81) | 0.178 |
| Genecology & obstetric | 11 (28.9) |  |  | 0.07 (0.01, 0.81) | 0.033 |
| Orthopedic | 19 (82.6) |  |  | [Reference] |  |
| Sex of Provider(Male) | 32 (88.9) |  |  | 1.18 (0.29, 4.88) | 0.817 |
| Length of stay (< 8 days) | 63 (66.3) |  |  | 0.78 (0.22, 2.80) | 0.702 |
| Experience (< 10 years) | 61 (67.0) |  |  | 1.94 (0.44, 8.50) | 0.378 |
