# Supplementary material for: Antibiotic usage in surgical prophylaxis: A prospective observational study in the surgical ward of Nekemte referral hospital
Source: PLoS One. 2018 Sep 13;13(9):e0203523. doi: 10.1371/journal.pone.0203523 (PMC6136737; doi:10.1371/journal.pone.0203523)
Supplement: S1 File — (DOCX) [file pone.0203523.s012.docx]

| **Surgery type** | **ASHP SAP recommendation** | **Ethiopian STG SAP recommendation** |
| --- | --- | --- |
| Gastro-duodenal | Cefazolin | Ciprofloxacin or  Cefazolin+ metronidazole |
| Biliary Tract | Cefazolin, cefoxitin, cefotetan, ceftriaxone, ampicillin–sulbactam | Ciprofloxacin or Cefazolin+ metronidazole |
| Appendectomy | (cefoxitin or cefotetan) or (cefazolin + metronidazole) | Cefazolin+ metronidazole |
| Small bowel | (cefoxitin or cefotetan) or (cefazolin + MTR) | Ciprofloxacin or  Cefazolin+ metronidazole |
| Hernia | Cefazolin | Cefazolin |
| Colorectal | Cefazolin + MTR, cefoxitin, cefotetan, ampicillin–sulbactam, ceftriaxone + metronidazole, ertapenem | Ciprofloxacin or  Cefazolin+ metronidazole |
|  |  |  |
|  |  |  |
| Gynecology and obstetrics | Cefazolin, cefotetan, cefoxitin, ampicillin–  Sulbactamh | Ceftizoxime or Cefazolin |
| Orthopedic surgery | Cefazolin | Cefazolin or Ceftizoxime |
| Urologic surgery | Fluoroquinolone, trimethoprim–sulfamethoxazole, cefazolin, cefoxitin, Cefazolin+ metronidazole, Cefazolin±aminoglycosides | Ciprofloxacin or Cefazolin |
| Head and neck | Cefazolin ± metronidazole, cefuroxime ± metronidazole, ampicillin–sulbactam | Cefazolin or Pencillin G |
